# Sensor Based on a Poly[2-(Dimethylamino)ethyl Methacrylate-*Co*-Styrene], Gold Nanoparticles, and Methylene Blue-Modified Glassy Carbon Electrode for Melamine Detection

**DOI:** 10.3390/s21082850

**Published:** 2021-04-18

**Authors:** Fairouz Aberkane, Imene Abdou, Nadia Zine, Nicole Jaffrezic-Renault, Abdelhamid Elaissari, Abdelhamid Errachid

**Affiliations:** 1 LCCE Laboratory, Department of Chemistry, Faculty of Matter Sciences, University Batna 1, Batna 05000, Algeria; imene.abdou@univ-batna.dz; 2 Institut de Sciences Analytiques (ISA)-UMR 5280, Université Claude Bernard Lyon 1, 5 Rue de la Doua, 69100 Lyon, France; nadia.zine@univ-lyon1.fr (N.Z.); nicole.jaffrezic@univ-lyon1.fr (N.J.-R.); Abdelhamid.Elaissari@univ-lyon1.fr (A.E.); abdelhamid.errachid-el-salhi@univ-lyon1.fr (A.E.)

**Keywords:** electrochemical sensor, gold nanoparticles, melamine, methylene blue, glassy carbon electrode, modified electrode, copolymer

## Abstract

Melamine has been used as a non-protein nitrogenous additive in food products to artificially increase the apparent “false” protein content. Melamine is known as a dangerous and poisonous substance for human health and it causes diverse diseases. An electrochemical sensor for melamine detection has been developed by modification of a glassy carbon electrode using copolymer poly[DMAEMA-*co*-styrene], gold nanoparticles, and methylene blue. The characterization of the modified electrode was conducted using several analysis techniques including cyclic voltammetry (CV), differential pulse voltammetry (DPV), chronoamperometry (CA), and electrochemical impedance spectroscopy (EIS). The electrochemical detection of melamine was performed by impedance spectroscopy. Obtained results revealed that the developed sensor has a large detection range from 5.0 × 10^−13^ to 3.8 × 10^−8^ M with a low detection limit of 1.8 × 10^−12^ M (at S/N = 3). Various interfering species such as phenol, hydroquinone, and bisphenol A have been used and their behavior on modified electrode has been studied.

## 1. Introduction

Quality control of our food and our environment (monitoring of industrial or domestic discharges) increasingly requires the development of precise and selective analytical methods [1,2,3]. Melamine (2,4,6-triamino-1,3,5-triazine) is an organic compound usually used in the preparation of thermosetting melamine-formaldehyde polymer resins, which are used in the composition of various plastics such as kitchenware, adhesives, cement, and flame retardant paints [4]. In the environment, melamine is considered as an organic micropollutant that can be released during the production process, and through migration from the degradation of polymers containing melamine and its derivatives [5,6]. Due to its low biodegradability, melamine may escape conventional wastewater treatment plants and as a consequence, it can be discharged into surface water [7]. Recent studies have reported detections of melamine in different matrices of water (river water (370 ng/L), lake water (347 ng/L), seawater (186 ng/L), tap water (512 ng/L), bottled water (98 ng/L)…) [8]. The migration of melamine into food occurs in measurable amounts, especially under acidic conditions and at high temperatures, and is also dependent on contact time [9]. On the other hand, melamine has sometimes been added as a non-protein nitrogenous additive to artificially increase the apparent “false” protein content in foodstuffs [10,11]. According to preliminary reports of adulterated infant milk, melamine has been found in several food products and detected in various items such sweets, drinks, and cookies [12]. 

However, exposure to melamine can be particularly hazardous for human health as melamine has been found to be toxic at high concentrations (≥2.560 mg/kg), which can cause severe kidney diseases and also lead to death in children and infants [13,14,15]. Melamine can undergo a hydrolysis reaction leading to cyanuric acid formation, which can associate with melamine molecules to form insoluble complexes. These complexes can produce crystals in the urinary system, eventually leading to grave kidney failure. Additionally, it causes various toxic effects on the kidneys such as inflammation, and can even produce bladder cancer [4,16]. The European Food Safety Authority as well as The World Health Organization have announced that the tolerable daily intake for melamine is 0.2 mg/kg of body weight per day [17,18]. 

Analytical chemistry including electroanalytical chemistry can play a very important role in the protection of human health and the environment by controlling the harmful effects of melamine. It is used to determine the amount of melamine employed in products (especially in food) to find out if the amount added does not exceed the doses beyond which melamine becomes toxic. Therefore, electroanalytical chemistry indirectly controls the harmful effects of melamine by monitoring the amount of melamine used. Electrochemical sensors constitute simple, reliable, rapid, and selective detection systems [19,20,21] and are very useful for on-site monitoring of pollutants [22,23,24]. Besides electrochemiluminescence sensors, based on the emitted light originated from excited states formed through the electron transfer reaction at the surface of the electrode [25], and the electrochemically printed polymer sensors based on the selective adsorption of melamine at the surface of the modified electrode [26,27], the use of different electrochemical probes to improve the poor electroactivity of melamine remains a very simple and effective way to achieve the indirect determination of melamine. Some strategies have been adopted based on the conversion of non-electroactive melamine into electroactive melamine complexes [28], or into an electroactive melamine-based polymer [29]. The hydrogen bonding recognition effect has also been used to determine melamine. Cao et al. investigated the use of ferricyanide as an electrochemical indicator to study, by voltammetric methods, the interaction between melamine and oligonucleotide modified gold electrodes. The results indicate that the electrostatic and hydrogen-bonding interactions between oligonucleotides and melamine lead to the increase in the peak currents of ferricyanide, which could be used for the electrochemical sensing of melamine [30]. Correspondingly, Liao et al. have used uric acid as an electrochemical probe to determine melamine concentration at the surface of pre-anodized screen-printed carbon electrodes (SPCE*). According to the authors, melamine and uric acid can bind to the SPCE* surface through hydrogen bonds, so there is a competitive adsorption behavior between melamine and uric acid. The adsorption of melamine to the surface of SPCE* results in the decrease of the uric acid oxidation current, therefore it can be used to build up an electrochemical detection scheme for the determination of melamine [31]. In Rovina et al.’s study, a novel electrochemical biosensor was developed based on the modification of gold electrode with chitosan, calcium oxide nanoparticles, and an ionic liquid for the determination of melamine. The electrochemical behavior of the modified gold electrode was studied in the presence of methylene blue as a redox indicator. The adsorption of melamine at the modified electrode surface leads to an increase in the oxidation peak current of methylene blue, where this increase is dependent on the melamine concentrations [32]. 

In the present work, an electrochemical sensor for melamine detection was developed by modification of a glassy carbon electrode using a crosslinked copolymer poly[2-(dimethylamino)ethyl methacrylate-*co*-styrene], gold nanoparticles, and methylene blue. Methylene blue was used as a modifier for the glassy carbon electrode surface for the improvement of melamine detection. The enormous specific surface area of gold nanoparticles due to their nanometric size facilitates the adsorption of a large amount of methylene blue at the modified electrode surface. The combination between the three compounds was used for the first time. The copolymer thus used was synthesized using radical emulsion polymerization. The characterization of the synthesized copolymer and the elaborated modified electrode was carried out by different techniques. Both the copolymer and methylene blue have heteroatoms and can create hydrogen-bonding interactions with melamine. The electrochemical detection of melamine was performed by impedance spectroscopy indirectly using an electrochemical probe. Several interfering species such as phenol, hydroquinone, and bisphenol A were used and their behavior on the modified electrode was studied. 

## 2. Materials and Methods

All chemicals needed for the experiments were of analytical purity and used as received. Tetrachloroauric acid (HAuCl_4_.3H_2_O, ≥99.999%), potassium hexacyanoferrate(II) trihydrate(>98.5%), potassium hexacyanoferrate(III) (>99%), 2-(dimethylamino)ethyl methacrylate (DMAEMA, 98%), styrene (≥99%) bisphenol A (BPA, ≥99%), melamine (MEL, ≥99%), 1,6-hexanediol diacrylate (HDDA, 80%), ammonium persulfate ((NH_4_)_2_S_2_O_8_, 98%), sodium dodecyl sulfate (SDS, ≥99%), methylene blue (MB, ≥97%) were obtained from Sigma Aldrich. Sodium tetrahydroborate (NaBH_4_, 98%) was purchased from Acros Organics. Hydroquinone (+99%) and phosphate buffered saline (PBS) were obtained from Sigma. Phenol (≥99.5%) was purchased from Riedel-DeHaën. Alumina powder (99.9%) was obtained from Aldrich. An aqueous 10^−6^ mol/L melamine solution was used as a stock solution.

Electrochemical experiments were realized with an Autolab potentiostat (Potentiostat-Galvanostat PGSTAT30, METROHM, France) controlled by two software packages General Purpose Electrochemical System (GPES) and Frequency Response Analysis (FRA) for Windows—version 4.9.005. All electrochemical experiments were conducted using a conventional three-electrode cell (50 mL) consisting of a glassy carbon electrode (GCE) with a 0.78 cm^2^ surface area as a working electrode, a platinum auxiliary electrode, and a silver chloride electrode (Ag/AgCl) as a reference electrode. All measurements were performed in 0.01 M PBS (pH 7.4) containing 5 mM Fe(CN)_6_^3−/4−^ ions at room temperature. The cyclic voltammetric analysis of the modified electrodes in the presence of Fe(CN)_6_^3−/4−^ was achieved in the potential ranging from −0.6 to 1 V with a scan rate of 100 V/s. The electrochemical impedance analysis was carried out in the frequency range between 2 kHz and 0.1 Hz at 0 V (amplitude equal to 0.005 V).

### 2.1. Characterization Techniques

Scanning electron microscopy (SEM) was used to study the morphology of the prepared copolymer particles. SEM was carried out at the Technological Center for Microstructures at the University of Lyon (Villeurbanne, France). A droplet of a dilute aqueous suspension of particles was placed on a flat steel support and dried at room temperature. Then, the copolymer sample was vacuum covered by spraying with platinum and observed by SEM at an accelerating voltage of 15 kV. 

Fourier transform infrared (FTIR) spectroscopy was used to identify the synthesized copolymer. The infrared spectrum was recorded on a PerkinElmer Two UATR spectro-photometer by attenuated total reflection on a diamond crystal in mono-reflection. The spectrum was from 4000 to 400 cm^−1^ by accumulating 20 scans with a resolution of 4 cm^−1^.

### 2.2. Synthesis of Poly(DMAEMA-Co-Styrene)

Poly(DMAEMA-*co*-styrene) (PN) crosslinked particles were prepared using radical emulsion polymerization. First, 0.6 mL of 2-(dimethylamino)ethyl methacrylate (DMAEMA) was dispersed in 30 mL of an aqueous solution of the SDS emulsifier (0.1 M). To this solution, 33.2 μL of 1,6-hexanediol diacrylate (HDDA) and 0.42 mL of styrene were added. Afterward, the reaction mixture was stirred until a clear solution was obtained. The radical polymerization reaction was initiated after the addition of 1 mL of the aqueous solution of the thermal initiator, (NH_4_)_2_S_2_O_8_ (1% *w/w*). The reaction proceeded with constant stirring (750 rpm) at 70 °C for 5 h. Finally, the copolymer was recovered by centrifugation (4000 rpm) and washed using methanol [33].

### 2.3. Synthesis of Modified Electrode GCE/PN-AuNPs-MB

**Scheme 1:**
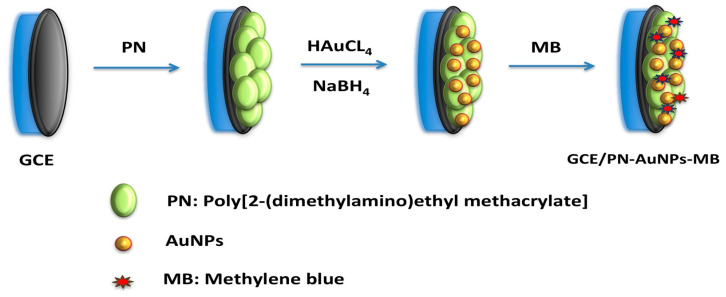
Preparation steps of the GCE/PN–AuNPs–MB modified electrode.

The modified electrode, GCE/poly(DMAEMA-*co*-styrene)—gold nanoparticles—MB (GCE/PN-AuNPs-MB), was prepared in three steps (Scheme 1). In the first step, the GCE was polished using alumina powder; a small quantity of powder was placed on a piece of carpet, and a few drops of deionized water were added. Then, the electrode was polished by circular motions until obtaining a mirror-like surface, then washed ultrasonically in water for 10 min. Thereafter, a 7-μL aliquot of PDMAEMA-*co*-styrene, dispersed in water, was applied to the clean GCE, before the electrode was left to air dry for 48 h. Next, the GCE/PN was incubated in 5 mL of 0.25 mM HAuCl_4_ solution for 10 min, then in 5 mL of 0.026 M of NaBH_4_ solution for 5 min. Finally, the electrode was placed in 5 mL of 0.125 mM MB solution for 5 min. 

## 3. Results and Discussion

### 3.1. Synthesis and Characterization of Poly(DMAEMA-Co-Styrene)

**Scheme 2:**
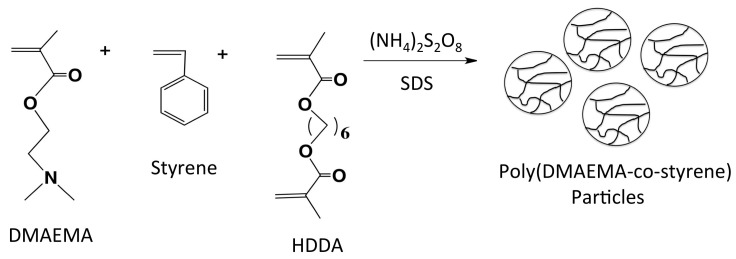
Reaction of radical emulsion copolymerization of styrene and DMAEMA in the presence of HDDA.

The PN copolymer was synthesized using emulsion polymerization. In this work, DMAEMA and styrene were used as co-monomers, while HDDA was used as a crosslinking agent. The stability of the micelles containing DMAEMA, styrene, and HDDA in water was ensured by the addition of the SDS surfactant. Then, the polymerization reaction was initiated using a hydrophilic initiator ((NH_4_)_2_S_2_O_8_, and the polymerization took place in three conventional steps of radical polymerization. Scheme 2 shows the overall reaction of the copolymerization. The final solution obtained after reaction was transparent and slightly yellow in color. The unreacted monomers and the remaining reactants were removed by multiple washings with methanol; the yield obtained was 78.46%.

**Figure 1:**
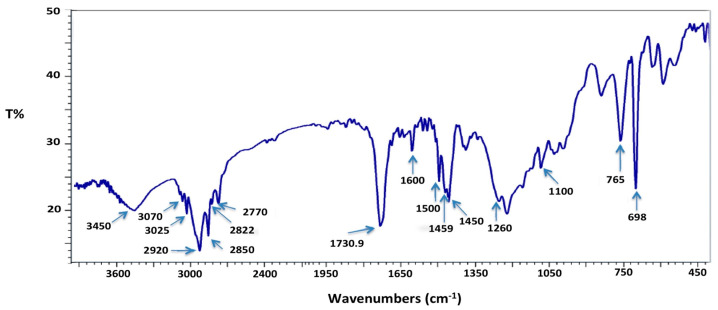
FTIR spectrum of poly(DMAEMA-*co*-styrene).

The Infrared spectrum (Figure 1) shows the characteristic bands for poly(DMAEMA-*co*-styrene). The absorption bands located at 3070 and 3025 cm^−1^ can be attributed to stretching vibrations of the aromatic C–H bonds. The three absorption bands at 1600, 1450, and 1500 cm^−1^ are due to the vibrations of the C=C double bonds of the benzene rings. The absorption bands at 698 cm^−1^ and 765 cm^−1^ correspond to the vibration of the C–H out of the plane. The absorptions at 2850, 2920, and 1459 cm^−1^ correspond to the existence of methyl and methylene groups. The absorption band at 3450 cm^−1^ can be attributed to the stretching vibrations of O–H bonds (traces of H_2_O). The characteristic bands located at 2770 cm^−1^ and at 2822 cm^−1^ correspond to the stretching vibrations of the (C–H) bonds in (N–CH_3_), while the bands appearing at 1730.9 cm^−1^ and 1100 cm^−1^ can be attributed to the stretching vibrations of the bonds (C=O) and O–C=O of the acrylate group and the absorption band at 1220 cm^−1^ corresponds to the C–N bond of the tertiary amino group [34,35].

**Figure 2:**
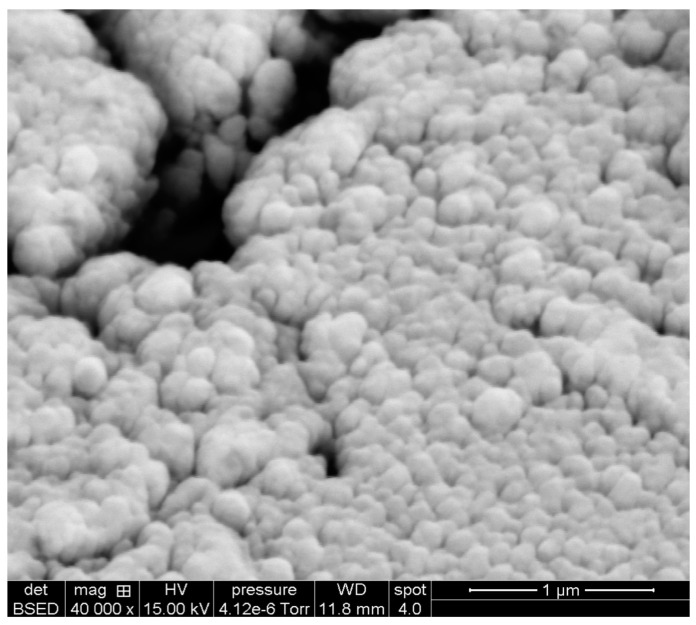
Scanning electron micrograph of poly(DMAEMA-*co*-styrene) particles.

SEM was also employed to study the morphology of the obtained polymer particles. The scanning electron micrograph depicted in Figure 2 shows that the synthesized copolymer was in the form of large spherical particles, which have different sizes. Each particle consisted of a grand number of small cauliflower-like, nano-sized polymer particles. The small particles have combined to form particles of larger sizes.

### 3.2. Electrochemical Characterization of Modified Electrode GCE/PN–AuNPs–MB 

The synthesized copolymer used to modify the GCE contains organic functions, which are useful for complexation with Au^+3^ ions before they were reduced by NaBH_4_, and for the formation and stabilization of AuNPs. Additionally, by forming hydrogen bonds with the particles of the copolymer, MB was easily adsorbed on the GCE to yield a uniform film. The surface modification of the GCE was monitored and analyzed by cyclic voltammetry and electrochemical impedance spectroscopy. 

**Figure 3:**
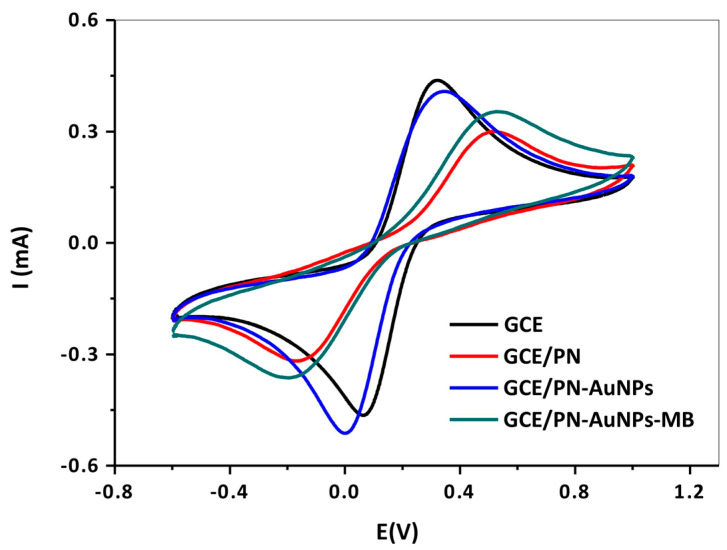
Cyclic voltammetry of: GCE, GCE/PN, GCE/PN–AuNPs, and GCE/PN–AuNPs–MB electrodes. The measurements were made in PBS containing Fe(CN)_6_^3−/4−^ in the potential ranging from −0.6 to 1 V (scan rate of 100 V/s).

The cyclic voltametric results of Fe(CN)_6_^3−/4−^ at the GCE, GCE/PN, GCE/PN–AuNPs, and GCE/PN–AuNPs–MB in the presence of Fe(CN)_6_^3−/4−^ are shown in Figure 3. A pair of intense redox peaks was observed for the GCE (anodic peak current Ia = 0.42 mA and cathodic peak current Ic = 0.44 mA) with a peak-to-peak separation ΔEp of 0.27 V. For the different modified electrodes, the obtained redox peaks of Fe(CN)_6_^3−/4−^ had lower intensities with a peak-to-peak separation ΔEp equal to 0.68 V for GCE/PN (Ia = 0.20 mA, Ic = 0.22 mA), and 0.75 V for GCE/PN–AuNPs–MB (Ia = 0.25 mA, Ic = 0.24 mA). However, the GCE/PN–AuNPs electrode presented the highest intensity Ia = 0.39 mA, and Ic = 0.47 mA with a ΔEp of 0.35 V. These results indicate that the conductivity of the modified electrode was improved in the presence of AuNPs compared to the modified electrodes without AuNPs; AuNPs promote charge transfer, which leads to an increase in current [36]. However, the presence of the polymer layer and MB molecules block the electron transfer at the electrode interface. 

**Figure 4:**
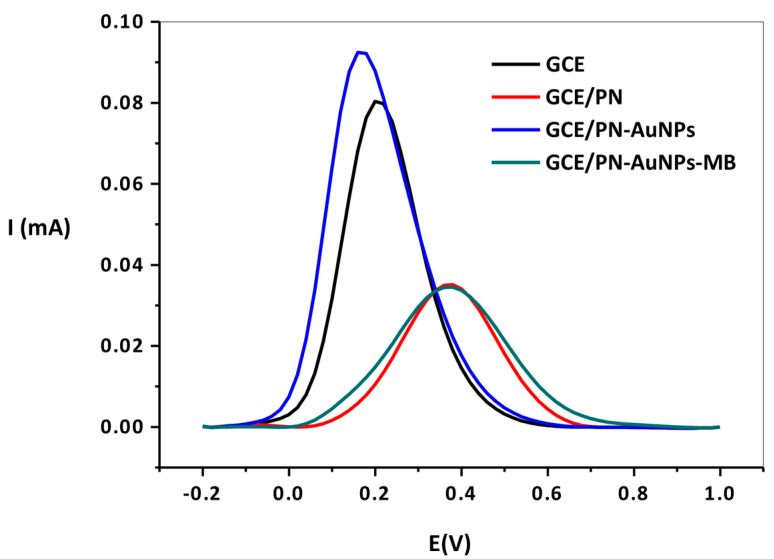
Differential pulse voltammograms of: GCE, GCE/PN, GCE/PN–AuNPs, and GCE/PN–AuNPs–MB electrodes. Signals measured at room temperature in PBS in the presence of Fe(CN)_6_^3−/4−^ (5 mM), scan potential from −0.2 to 1 V (pulse amplitude: 0.025 V; Pulse period: 0.2 s; Pulse width: 0.05 s).

Differential pulse voltammograms of 5 mM [Fe(CN)_6_]^3−/4−^ at a GCE, a GCE/PN, GCE/PN–AuNPs, and a GCE/PN–AuNPs–MB in PBS are shown in Figure 4. A typical signal of 80.3 μA was generated by the Fe(CN)_6_^3−/4−^ redox couple reaction at the surface of the GCE. This signal was found to have decreased to 35.1 μA after PDMAEMA-*co*-styrene was immobilized. We attributed this decrease in current to a loss of conductivity at the copolymer-coated electrode. Meanwhile, this peak current increased to 92.5 μA after incorporating AuNPs in the PDMAEMA-*co*-styrene layer on the electrode. This increase is due to the catalytic effect of AuNPs in improving the transfer of electrons of Fe(CN)_6_^3−/4−^. On the other hand, the peak current was observed to decrease to 34.5 μA at GCE/PN–AuNPs–MB because of an inhibition effect on the electron transfer of MB. The differences observed in the DPV curves were the result of different materials deposited on the GCE surface.

**Figure 5:**
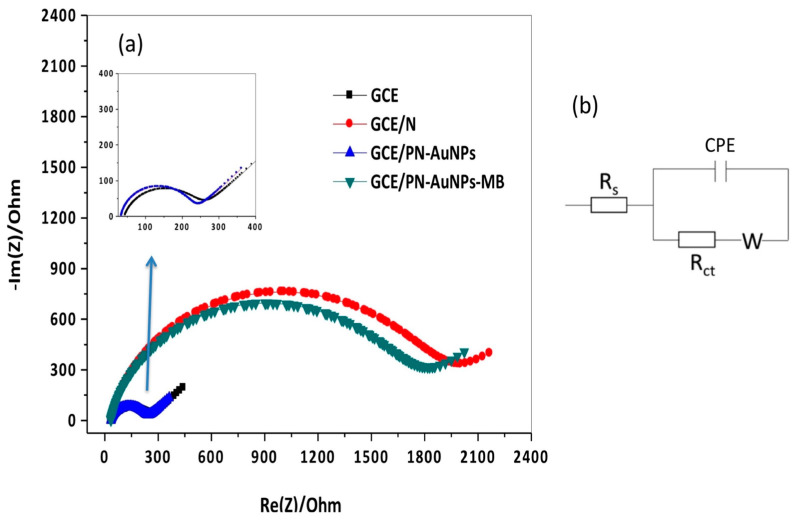
(**a**) Nyquist plots of 5 mM of Fe(CN)_6_^3−/4−^ at a GCE (black), GCE/PN (red), GCE/PN–AuNPs (blue), and GCE/PN–AuNPs–MB (green). The measurements were carried out in a 0.01 M PBS (pH 7.4) supporting electrolyte. The frequency was varied from 2 kHz to 0.1 Hz at a DC potential of 0 V and a peak-to-peak amplitude of 0.005 V for the AC potential, the insert is an enlargement of the impedance spectra of the GCE and the GCE /PN−AuNPs. (**b**) Randles model.

Figure 5a shows the impedance spectra of 5 mM [Fe(CN)_6_]^3−/4−^ at a GCE, a GCE/PN, GCE/PN–AuNPs, and a GCE/PN–AuNPs–MB. The electrochemical impedance behavior of the electrodes was adapted to the Randles model (Figure 5b), where “Rs” is the solution resistance, “Rct” is charge transfer resistance, “CPE” is the constant phase element, and “W” represents Warburg impedance. The (imaginary impedance -Im(Z) versus real impedance Re(Z)) Nyquist plot obtained at the bare GCE (Black trace) showed a small semicircle with a diameter of 163 Ω, which was used as an estimate of the charge transfer resistance. At the GCE/PN, the charge transfer resistance increased to 1795.9 Ω. After incorporating AuNPs on the GCE/PN, the charge transfer resistance decreased to 161.3 Ω. This was attributed to the good catalytic effect of AuNPs as they have an enormous specific surface area due to their nanometric sizes, which involve a larger contact surface with the surrounding medium (the electrolyte), leading to an important charge transfer. However, immobilization of the MB molecules on the GCE/PN–AuNPs surface electrode led to an increase to 1630.6 Ω, most likely due to the poor conductivity of MB. Using Equation (1) [37], the coverage of the GCE/PN–AuNPs–MB was calculated (θ_1_ = 90%).

(1) $$\theta_{1}=1-\frac{R_{CT0}}{R_{CT}}$$

where R_CT0_ represents the charge transfer resistance of the bare GCE and R_CT_ represents the charge transfer resistance of the GCE/PN–AuNPs–MB under the same condition.

**Figure 6:**
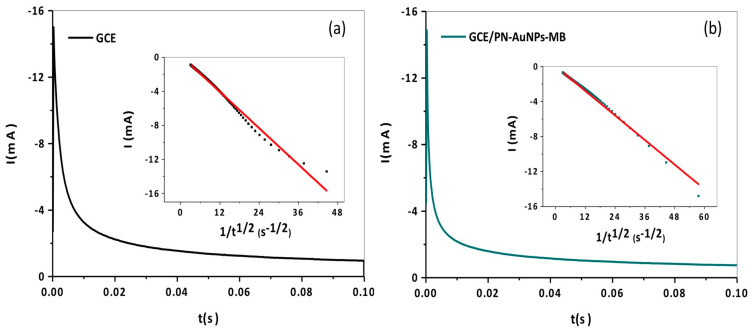
The curves (I − t) of: (**a**) GCE and (**b**) GCE/PN−AuNPs−MB and the plots of the curves (I − t^−1/2^) derived from the corresponding chronoamperometry data. Chronoamperometric measurements were carried out at a potential of 0.2 V in PBS containing Fe(CN)_6_^3−/4−^ ions (5 mM).

The electrochemical active surface of the modified electrodes was determined by chroamperometry as previously described [38]. Briefly, two potentials 0.2 V and 0.5 V were applied to GCE and GCE/PN–AuNPs–MB, respectively, corresponding approximately to the highest current intensity obtained by cyclic voltammetry for the two electrodes (black and green curves in Figure 3), and the current was recorded as a function of time, based on Cottrell’s equation in Equation (2), where n is the number of electrons involved, F is the Faraday constant (96,500 C/mol), A is the electrochemical active surface, C is the concentration of Fe(CN)_6_^3−/4−^ (5 mM), and D is the diffusion coefficient (7.6 × 10^−6^ cm^2^ s ^−1^) [39]. Accordingly, the slope of an I versus (t)^−1/2^ plot will yield an estimate for the electrode surface area, A. The plots constructed using results obtained at a GCE and at a GCE/PN–AuNPs–MB, respectively, are shown in Figure 6. The electrochemical active surface of the GCE before and after surface modification by PN, AuNPs, and MB was determined using Cottrell’s equation (Equation (2)) and the slope of the curve I = f (t^−1/2^). The two plots in Figure 6 are linear between 3.16 and 30.15 s^−1/2^ in the plot for GCE and between 3.16 and 44.72 s^−1/2^ in the plot for GCE/PN–AuNPs–MB, which can be respectively represented by the expression I (t) = 2.14 × 10^−4^ − 3.56 × 10^−4^ * t ^−1/2^ (R^2^ = 0.9913) and I (t) = 3.65 × 10^−5^ − 2.34 × 10^−4^ * t^−1/2^ (R^2^ = 0.9932). In this way, the electroactive surface of GCE and GCE/PN–AuNPs–MB were estimated as 0.47 cm^2^ and 0.32 cm^2^, respectively, based on a D value of 7.6 × 10^−6^ cm^2^ s ^−1^ adopted from [39] with similar experimental conditions. By comparing the two values, the electrochemical active surface decreased; the (PN–AuNPs–MB) is an inhibitory layer, causing a decrease in the transfer of electrons between the surface of the GCE/PN–AuNPs–MB and the electrolyte.

(2) $$I_{\left(t\right)}=nFAC\sqrt{\frac{D}{\pi t}}$$

### 3.3. Electrochemical Behavior of Melamine on the GCE/PN–AuNPs–MB

**Figure 7:**
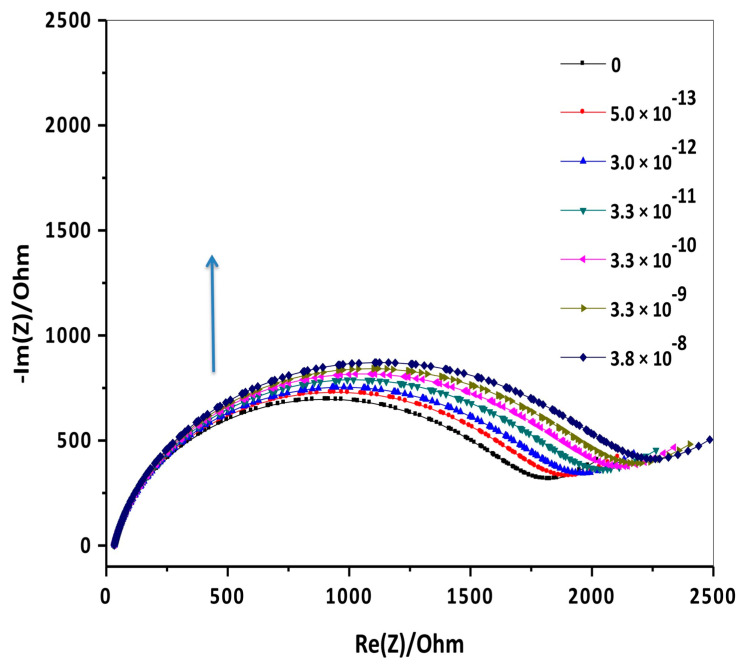
Nyquist curves of the electrochemical detection of MEL. The analysis was carried out at 0 V in the frequency ranging from 2 kHz to 0.1 Hz (Amplitude 0.005 V).

Electrochemical impedance spectroscopy is a powerful electrochemical technique. Being non-destructive, relatively sensitive, and selective, EIS has interesting fields of application in water and food analysis [40,41]. The electrochemical detection of Melamine (MEL) on the prepared modified electrode (GCE/PN–AuNPs–MB) was evaluated by electrochemical impedance spectroscopy. Solutions containing MEL at different concentrations between 5.0 × 10^−13^ and 3.8 × 10^−8^ M were analyzed. The analysis was carried out after an accumulation of 30 s for each concentration by scanning the potential from −0.2 to 1 V at a scanning rate of 100 mV/s. The accumulation was done under stirring (750 rpm). Impedance measurements were performed in PBS containing Fe(CN)_6_^3−/4−^ ions in the frequency ranging from 2 kHz to 0.1 Hz at 0 V. At this equilibrium potential, the current is approximately equal to 0 (according to Figure 3), therefore we can observe the electrochemical phenomena without disturbing the system, and study the impedance only by applying the sinusoidal signal. Figure 7 shows the impedance responses at the GCE/PN–AuNPs–MB for different MEL concentrations. The first semicircle of the Nyquist diagram corresponds to the electrochemical response of the GCE/PN–AuNPs–MB in the absence of MEL. After each addition of MEL, the semicircle diameter of the Nyquist plot increased, indicating an increase in R_CT_ (see Figure 7), which could have arisen from the adsorption of the non-conductive MEL on the electrode surface. The resulting impedance diagrams, in the presence of different MEL concentrations, were modeled by conducting a computer simulation using the equivalent electrical circuit illustrated in Figure 5b (Randles circuit). 

**Figure 8:**
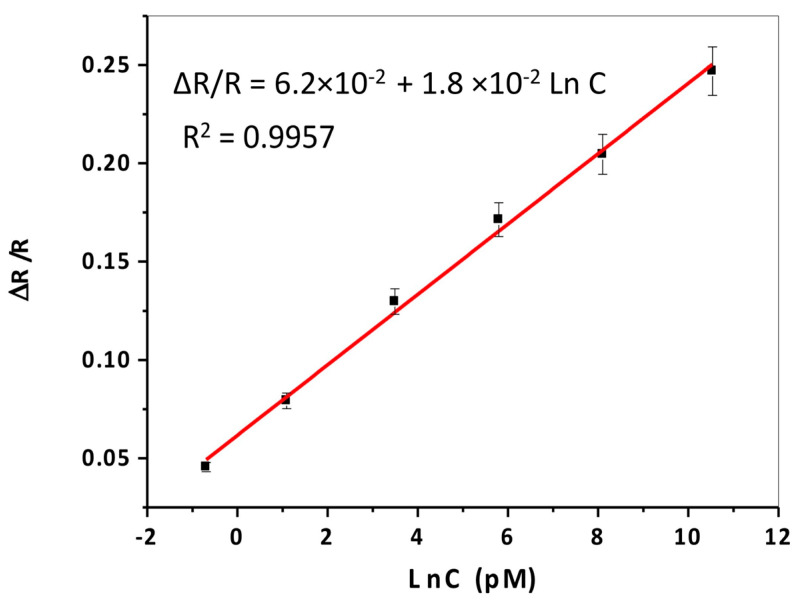
Calibration plot of GCE/PN–AuNPs–MB sensor for MEL detection.

The electrochemical response of MEL at the GCE/PN–AuNPs–MB shows a linear relationship with MEL concentration. Figure 8 shows that the response signal ΔR/R varies linearly with the logarithm of the MEL concentrations (ΔR/R = (R_CT1_ − R_CT_)/R_CT_), where R_CT_ and R_CT1_ represent the charge transfer resistance of the GCE/PN–AuNPs–MB in the absence and the presence of MEL with different concentrations, respectively). Here, ΔR/R was plotted to normalize the obtained signal and to have exploitable information. In this case, a comparison between several electrodes could be established. On the other hand, the curve with R_CT_ changed from one electrode to another because of the uniformity of the surfaces of the electrodes, with ΔR/R, we eliminated the differences in the thickness of the sensitive layer deposited on the surface of the electrodes. In the concentration range between 5.0 × 10^−13^ M and 3.8 × 10^−8^ M, the regression equation is ΔR/R = 6.2 × 10^−2^ + 1.8 × 10^−2^ Ln C, with a coefficient of determination R^2^ = 0.9957 (N = 6). The detection limit (LOD) of MEL is calculated using the equation LOD = 3.3 SD/b, where (SD) and (b) are the standard deviation of the intercept and the slope of the calibration plot, respectively [42], and the calculated LOD is equal to 1.8 × 10^−12^ M (at S/N = 3). Moreover, the reproducibility of the modified electrode GCE/PN–AuNPs–MB was assessed by the measurement of the response of three different modified electrodes, prepared with the same modification, to a 3 × 10^−12^ M of MEL solution, by impedance spectroscopy. The relative standard deviation calculated was 9.1%. Modified electrodes used for MEL detection and their characteristics, in a previous work, are presented in Table 1. 

The standard additions method was performed to test the effectiveness of the GCE/PN–AuNPs–MB in determining MEL concentration in deionized water samples. Two aqueous solutions of the MEL were prepared with a concentration of 1 × 10^−11^ M and 5 × 10^−12^ M in PBS containing Fe(CN)_6_^3−/4−^ (at a concentration of 5 mM). The standard additions of the MEL were made by adding different concentrations of MEL to these two samples [48,49]. After pre-concentration of the modified electrode in these solutions, the Nyquist diagrams were recorded under the same conditions. The results obtained through the standard addition method are in good agreement with the value of MEL added in the samples (Table 2).

### 3.4. Selectivity of the GCE/PN–AuNPs–MB

**Figure 9:**
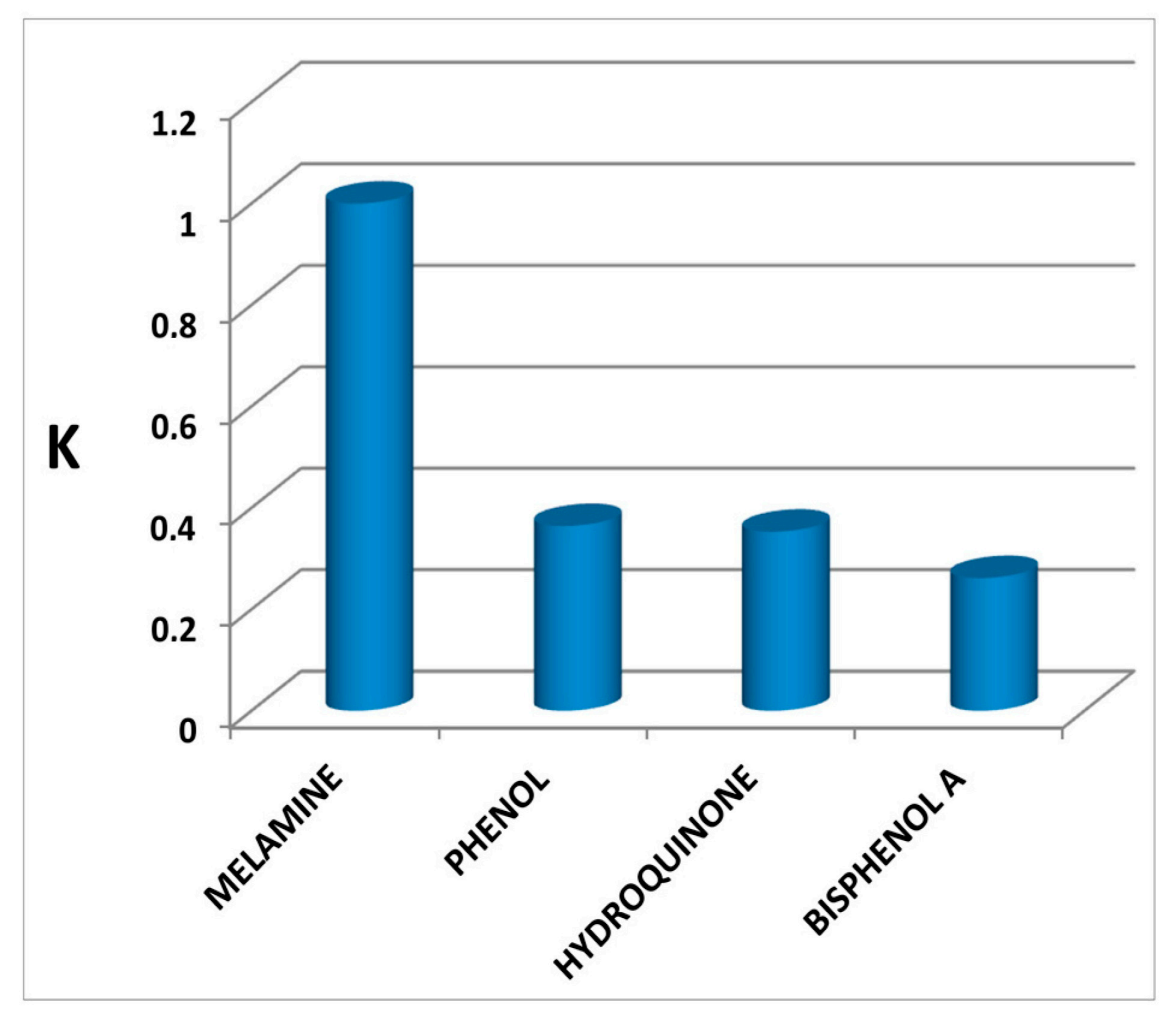
Selectivity of GCE/PN–AuNPs–MB for MEL relative to phenol, hydroquinone, and BPA at the concentration of 3.3 × 10^−9^ M.

The selectivity of the developed modified electrode was checked. Interfering products such as phenol, hydroquinone, and, BPA were used and their behavior on the modified electrode was studied. The choice of interfering agents was based on the location of the analyte in daily life. MEL is used as a monomer in polymer production, which is introduced in the composition of several plastics, in particular, in kitchenware. In general, plastic can release, besides MEL, products such as BPA, phenol, hydroquinone, or resorcinol, which are generally used as stabilizers. Under optimal conditions, the interference assay was performed in the presence of the same concentration (3.3 × 10^−9^ M) of MEL, hydroquinone, phenol, and BPA. A selectivity factor K was calculated for each interferent using Equation (3), where ΔR/R = (R_CT2_-R_CT_)/R_CT_, R_CT2_ is the value of charge transfer resistance after incubation with the concentration of analyte and R_CT_ is the value recorded for the modified electrode before incubation with the analyte (as above-mentioned). The results, presented in the form of a histogram (Figure 9), show that the elaborated sensor has no sensitivity for these organic compounds compared to MEL.

(3) $$K=\frac{∆R/R\left(interferent\right)}{∆R/R\left(melamine\right)}$$

### 3.5. Mechanism and Adsorption Energy of MEL on GCE/PN–AuNPs–MB

**Figure 10:**
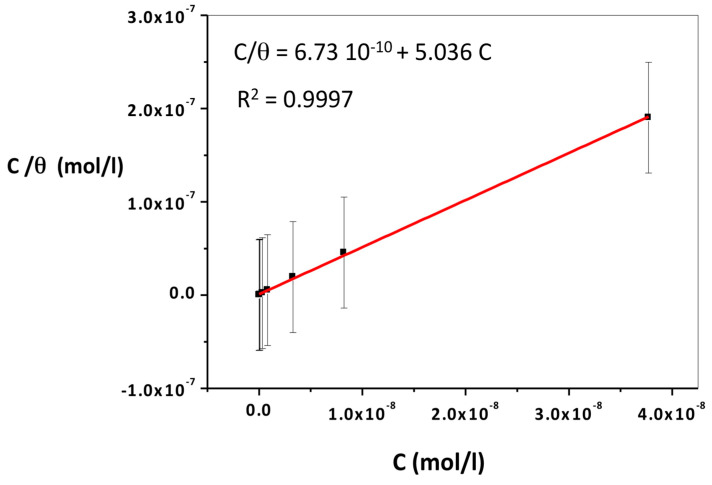
Langmuir isotherm of MEL adsorption on the GCE/PN–AuNPs/MB.

The mechanism of adsorption of MEL, at the surface of the GCE/PN–AuNPs–MB, was determined as in our previous work [38]. To understand the mechanism of adsorption, a covering area θ was calculated from the impedance results obtained for each concentration of MEL, in the same range of concentration studied, by applying Equation (4). The covering area increased as the amount of MEL adsorbed on the surface of the modified electrode increased. The results were fitted based on several adsorption isotherms including the Langmuir isotherm [50]. All fitting results are tabulated in Table 3. After analysis of these results, the Langmuir model (Equation (5), where C is the MEL concentration and K_Ads_ is the equilibrium constant of the chemical adsorption process) gives the best linearity (R = 0.9998) with the lowest p-value in comparison with the other isotherms, so it seems to be the most suitable. The curve of C/θ as a function of C is a straight line (Figure 10), therefore, the adsorption of MEL obeys the isotherm of Langmuir. Using Equation (6), where ΔG° is the free energy of adsorption, T and R are the absolute temperature and the ideal gas constant, respectively, (55.5 × 18) is the mass concentration of water in g L^−1^, the adsorption free energy was found to be −53.75 kJ/mol with an equilibrium constant equal to 1.485 × 10^9^ L/mol, which confirms that the type of adsorption of MEL on the modified electrode is chemical. MEL contains several nitrogen atoms in its structure and can easily form hydrogen bonds with any heteroatoms present on the surface of the GCE/PN–AuNPs–MB.

(4) $$\Theta =1-\frac{R_{\mathrm{CT}}}{R_{\mathrm{CT}1}}$$

(5) $$\frac{C}{\Theta }=\frac{1}{K_{\mathrm{ads}}}+C$$

(6) $$\Delta G{}^{\circ}=-\mathrm{RT}\;\mathrm{Ln}\;55.5\times 18K_{\mathrm{Ads}}$$

## 4. Conclusions

In the present work, an impedimetric sensor for MEL detection was developed, based on the modification of a GCE. Modification of GCE was carried out using a PN copolymer, AuNPs, and MB. The copolymer was synthesized by radical emulsion polymerization and characterized by FTIR and SEM, while the modified electrode was characterized with CV, DPV, CA, and EIS. The obtained results show that the elaborated sensor has a large detection range from 5.0 × 10^−13^ to 3.8 × 10^−8^ mol/L with R^2^= 0.9957 (N = 6), and the detection limit was found to be 1.8 × 10^−12^ mol/L (at S/N = 3). The presence of gold nanoparticles, at the surface of the modified electrode, leads to charge transfer activation, while, the addition of MB improves the retention of melamine at the surface of the modified electrode. Besides, MEL adsorption energy and the corresponding adsorption constant were determined using impedance results by the Langmuir isotherm. The adsorption free energy was found to be −53.75 kJ/mol with a high equilibrium constant equal to 1.485 × 10^9^ L/mol, which confirms that the type of adsorption of MEL on the modified electrode is chemical. Additionally, the selectivity of the modified electrode was confirmed by testing the modified electrode with several interfering molecules.

## Figures and Tables

**Table 1:** Comparison, based on the literature, in terms of linear range and limit of detection, between modified electrodes, used in the chemical detection of MEL, and the modified electrode developed.

Electrode	Method	Linear Range	LOD	Refs
Ionic liquid/calcium oxide nanoparticles/chitosan modified gold electrode	DPV	9.6 × 10^−3^ – 9.6 × 10^−15^ M	9.6 × 10^−16^ M	[32]
Glassy carbon electrode coated with a multi-wall carbon nanotube/chitosan composite	DPV	9.9 × 10^−9^ – 1.9 × 10^−7^ M	3.0 × 10^−9^ M	[43]
Gold nanoparticles deposited on a graphene doped carbon paste electrode	DPV	2 × 10^−10^ – 8 × 10^−7^ M and 8 × 10^−7^ – 8 × 10^−3^ M	1.8 × 10^−11^ M	[44]
Molecularly imprinted polypyrrole and graphene modified glassy carbon electrode	DPV	3 × 10^−8^ – 1 × 10^−4^ M	1.02 × 10^−8^ M	[45]
Molecularly imprinted poly(2-mercaptobenzimidazole) modified gold electrode	Impedance	1 × 10^−8^ M – 5 × 10^−5^ M	3 × 10^−9^ M	[46]
Ordered mesoporous carbon modified glassy carbon electrode	DPV	1 × 10^−8^ – 5 × 10^−7^ M	2 × 10^−9^ M	[47]
GCE/PN–AuNPs–MB	Impedance	5.0 × 10^−13^ − 3.8 × 10^−8^ M	1.8 × 10^−12^ M	This work

**Table 2:** Determination of MEL in deionized water samples.

Samples	Added (M)	Found (M)	Recovery Rate (%)
1	1 × 10^−11^	0.90 × 10^−11^	90
2	5 × 10^−12^	4.45 × 10^−12^	89

**Table 3:** Adsorption isotherm formulas, slopes and R (N = 6) of MEL adsorbed on the GCE/PN–AuNPs–MB.

Isotherm	Equation	Slope	R	*p*-Values
Langmuir	C/θ = 1/K + C	5.03	0.9998	3.0 × 10^−8^
Freundlich	Log θ = LogK + 1/nLogC	0.12	0.9371	2.9 × 10^−3^
Langmuir Freundlich	Log(θ/1 − θ) = f(LogC)	0.15	0.9279	3.8 × 10^−3^
Frumkin	Log(θ/(1 − θ)C) = LogK + 2a θ	−27.04	0.9803	2.9 × 10^−4^
Florry–Huggins	Log(θ/C) = f(1 − θ)	27.54	0.9809	2.7 × 10^−4^
Temkin	θ = lnC	0.013	0.9891	8.8 × 10^−5^
